# Effects of individual and dyadic decision-making and normative reference on delay discounting decisions

**DOI:** 10.1186/s41235-022-00422-5

**Published:** 2022-07-28

**Authors:** Diana Schwenke, Peggy Wehner, Stefan Scherbaum

**Affiliations:** Department of Psychology, Technische Universität Dresden, Zellescher Weg 17, 01069 Dresden, Germany

## Abstract

The tendency to devaluate delayed rewards, a phenomenon referred to as ‘discounting behaviour’, has been studied by wide-ranging research examining individuals choosing between sooner but smaller or later but larger rewards. Despite the fact that many real-life choices are embedded in a social context, the question of whether or not social collaboration can have an impact on such choices has not been addressed empirically. With this research, we aimed to fill this gap experimentally by implementing a novel choice selection procedure in order to study the interactive dynamics between two participants. This selection procedure allowed us to dissect the sequence of decision-making into its elements, starting from the very first individual preference to the solution of possible conflicting preferences in the dyad. In Experiment 1, we studied group decision-making on classical intertemporal choices to reveal the possible benefit of social collaboration on discounting and identified that the knowledge of the social situation in collective decision-making causes a reduction in discounting. In a pre-registered Experiment 2, we compared classical intertemporal choices with choices in a gamified version of a discounting paradigm in which the participants had a real-time experience trial by trial and for which a normative reference was present. We found that collective decision-making had a substantial impact on intertemporal decision-making, but was shaped by different types of choices. Classical intertemporal choices were rather susceptible to the contextual factors of decision-making, whereas in the gamified version that included a normative reference the decisions were reliably influenced by social collaboration and resulted in a lower discounting. The results in this paradigm replicate our original findings from former research.

**Supplementary Information:**

The online version contains supplementary material available at 10.1186/s41235-022-00422-5.

## Significance statement

In everyday life, we often feel torn between short-term enjoyment and long-term benefits, i.e. sooner but smaller or later but larger rewards. Such decisions, e.g. whether we are spending money now and buying a new mobile phone or saving money for pension or making impulsive food-related choices, are of high significance when we consider the implications of our choices on our personal, social and economic welfare, i.e. the financial situation of millions of people in retirement or the problem of overweight and obesity that can lead to serious health issues.

As humans we have developed a generic strategy to conquer our unwanted temptations: we consult an adviser; we create binding agreements or organize support groups, e.g. friends who are collectively trying to lose weight. To put it in a nutshell, we make these choices together with other people rather than alone. It is surprising that despite the fact that a large number of everyday future-related decisions are made together in groups, the question of whether discounting choices can benefit from social collaboration has been largely overlooked by experimental research. Therefore, we here aim to study whether joint decision-making can have a significant impact on the impulsiveness of delay discounting. Further, we question through which process this possible variation may occur and which moderating factors might influence the interactive processes and, in the end, the decision outcome.

## Introduction

Choices between a smaller reward available earlier and a larger reward available later are called intertemporal decisions and are ubiquitous in our everyday lives and in research. Although in reality many of these decisions are made jointly with a partner or in groups, there is little research on whether social collaboration influences intertemporal decisions. This is especially surprising since empirical studies have already shown that being embedded in a social context and making decisions together with others influences choices. In our previous research, using a gamified approach of an intertemporal choice task, in which the participants had a real-time experience trial by trial and for which a normative reference was present, we have already shown that social collaboration with a partner also influences intertemporal decisions. In our current study, we aim to investigate whether this finding can also be generalized to classical intertemporal decisions.

### Research on intertemporal decisions

Research on intertemporal decisions generally assumes that the value of the option at hand is discounted by the delay after which it becomes available. The tendency to devaluate delayed rewards has been studied widely, examining individuals choosing between monetary options, i.e. choosing between sooner but smaller (SS, e.g. 5€ in 1 day) or later but larger (LL, e.g. 10€ in 7 days) rewards. It has been shown that normative models that assume linearity over time (Samuelson, 1937) could not account for the usual behavioural patterns of human discounting. In fact, people tend to devalue an option’s value by the delay of its delivery, but this devaluation is stronger for early intervals, i.e. the subjective value of an option decreases more steeply than predicted, especially for immediate rewards (see Frederick et al., 2002 for a review). However, the extent of this discounting varies: some people turn down a large pay-out that is available later in favour of a smaller but sooner option while other people choose the opposite. This variation is often used as a proxy for persons’ impulsiveness, as strong discounting correlates with self-reported impulsiveness (Crean et al., 2000), dysfunctional behaviour like drug abuse (Amlung et al., 2017; Bickel et al., 1999; Coffey et al., 2003; Kirby et al., 1999; Petry, 2001a; Scherbaum et al., 2018), gambling (Alessi & Petry, 2003; Petry, 2001b), and overweight (see Sweeney & Culcea, 2017 for a review). Research on age differences also showed that children demonstrated a stronger orientation towards sooner but smaller choices and showed less anticipation of the future, indicating that the ability of impulse control follows different developmental timetables (Scheres et al., 2014; Steinberg et al., 2009). Studies that provided these results focused primarily on the determinants of individual discounting. However, many of the intertemporal choices of real life, especially those with high impact, are made together with others, e.g. spouses or teams. Regardless, the question of how single individuals decide on intertemporal choices has been the focus of research in the last decades.

### Influence of social contexts on intertemporal decisions

This is all the more surprising, as some studies impressively demonstrate that even embedding a decision in a social context can strengthen our ability to resist short-term temptations. Firstly, research on surrogate decision-making shows that participants were more likely to choose later but larger choice options, and therefore, their discounting functions were less steep when making decisions for somebody else than when making decisions for themselves (Albrecht et al., 2011; Batteux et al., 2017), an effect that declines with larger social distance between the deciding participant and the actual recipient of the choices(Pronin et al., 2008; Yi et al., 2011; Ziegler & Tunney, 2012). Further evidence suggests an increase in neural activity in dopaminergic systems for choice sets with an immediate choice option, but only if choices were made for oneself but not in terms of surrogate decision-making (Albrecht et al., 2011). Secondly, research on the influential effects of a social context on the impulsivity of discounting suggests an impact of observing other people’s choices. It has been shown that participants tend to even higher discounting after observing other people choose immediate instead of delayed choice options (Gilman et al., 2014). In a similar way, short-sighted subjects modified their choice responses towards less impulsive decision-making after observing low discounting behaviour trail by trial and vice versa (Calluso et al., 2017). The context of group decision-making also alters discounting decisions in such a way that individual participants were more patient, i.e. showed a decreased discounting, when their individual decisions were also applied to a hypothetical group of other recipients (Charlton et al., 2013). While these results clearly demonstrate an influence of a social context on individual discounting preferences, less research has targeted in particular the impact of collective decision-making on discounting.

### Group decision-making

One study that examined intertemporal decision-making in groups was conducted by Bixter et al. (2017). They investigated the contagion effect on discounting choices in a collaborative paradigm in which the participants completed three phases of a delay discounting paradigm, an individual pre-group and a post-group phase as well as an intervening group decision-making phase. During the collaboration phase, groups averaged the individual preferences of their members, whereas individual choices in the post-group phase converged towards the respective group choices (see also Bixter & Rogers, 2019). The fact that there are very rare studies of intertemporal decision-making is particularly surprising in the light of a whole body of literature on the effects of group decision-making in other areas. Here, it was shown that groups often overcome flawed, impulsive or ineffectively decision-making compared to the average performance of the individual members (for a review see Kerr & Tindale, 2004; Kugler et al., 2012) in e.g. reasoning (Cooper & Kagel, 2005; Maciejovsky et al., 2013), quantity estimations (Gigone & Hastie, 1997; Laughlin et al., 2003), perceptual discrimination (Bahrami et al., 2010). Different processes are postulated in order to explain this effect of group superiority. One line of research argues that the mere presence of other people leads to a higher individual task performance, referred to the so-called effect of “social facilitation”. Originally studied in cognitive perception tasks (Cottrell et al., 1968; Henchy & Glass, 1968; Zajonc, 1965), social facilitation was also observed to strengthen peoples’ regulatory abilities in terms of a lower level of impulsiveness (Herman, 2015; Herman et al., 2003; Roth et al., 2001; VanDellen & Hoyle, 2010). Since strong discounting is associated with low inhibitory abilities and high impulsiveness, as laid out above, social facilitation therefore may serve as a very strong influence on impulsive responses to alternative discounting options. However, another line of research highlights the beneficial effects of group-related social combination processes (Laughlin, 1980; Laughlin & Ellis, 1986). Due to the fact that groups have access to a larger pool of cognitive resource, information, and areas of expertise (Baumann & Bonner, 2004; Hinsz, 1990), group members are able to e.g. correct errors mutually (Bahrami et al., 2010), distribute different task demands (Wahn et al., 2017), or improve their individual performances through interactive group-to-individual learning (Maciejovsky et al., 2013). To put it briefly, groups can benefit from the exchange of information. At first glance, this interchange between group members in group decision-making tasks is especially useful in cognitive tasks that have a correct solution to a given problem. Classical discounting, however, does not operate on the basis of such a clear classification of correct vs incorrect, because preferences for sooner smaller options are not necessarily dysfunctional (Daly & Wilson, 2005). Therefore, it is an open question whether the exchange of information between group members in joint decision-making also provides a benefit in discounting tasks.

### Our previous research on intertemporal decision-making in groups

Nevertheless, we found some indication for group related exchanging processes in our own former research as we started to approach the influence of joint decision-making on discounting in a gamified delay discounting paradigm (Schwenke et al., 2017). Here, participants executed a series of choices between two delayed choice options in an individual (*individual decision*) and in a joint decision-making condition (*dyadic decision*) in a non-verbal choice selection procedure. In the individual condition, each participant moved a courser into an option associated response box via joystick movement. In the dyadic decision, both joystick movements were added up, so that both participants controlled the cursor together and had to coordinate in order to reach a mutual decision. Importantly, the cursor only started moving after both participants had indicated their first initial choice preference. With this procedure, we have disentangled the final dyadic decision from the initial first preferences of each participant (*pre-decision*). This individual decision was made before the partner’s preference in the current trial was known. In line with our assumptions, we found that participants discounted less in the dyadic condition compared to their average individual discounting. Furthermore, we studied the concrete process by which this variation could occur. Based on the segmentation into three distinguishable levels of decision-making (*individual decision, pre-decision, dyadic decision*), we found no significant difference between the individual decision in the individual condition and the initial pre-decision in the dyadic decision-making which would have indicated that the individual decision-maker in the dyadic condition had already changed his preference even bevor the interaction could take place. In contrast, we found significant differences between the pre-decision and the dyadic decision, which indicated that the interactive coordination between both participants had crucial influence on the final dyadic decision. From this we concluded that the lower dyadic discounting in the final dyadic decision is due to the interactive coordination rather than the social context itself. Our original approach strongly focussed on observing the decision-making process procedure in greater detail, which led us to use a gamified approach in this first study. However, this approach clearly differed from classical discounting tasks in terms of several characteristics. First and most importantly, participants collected the preferred rewards in real time instead of choosing between rewards in a distant future: in each trial, they had to wait the respective time and watch the avatar collecting the reward. Furthermore, they actually gained money based on their real choices in each trial. Classical discounting research in contrast operates with much larger rewards and delays, which are usually presented in hypothetical decision-making situations or in settings, in which one or a few choices are randomly chosen from the full set of all choices made and paid out at the respective time (Kaplan et al., 2016; Myerson et al., 2014). Another important difference is that our procedure provided the key benefit of an objective classification of each choice in being either an optimal or non-optimal decision according to a normative reference, a feature that has similarities with other, more cognitive group-decision-making tasks (Kerr & Tindale, 2004; Kugler et al., 2012). By implementing a normative reference, we separated the subjective devaluation from the objective efficiency of the decision which contrasts classical delay discounting studies that solely focus on the amount of subjective devaluation. Taking these differences into account, it is unclear whether our findings are applicable to classical intertemporal choices and a test of generalization is strongly indicated.

### Research goals

We hence present two studies investigating this question of generalization. In Study 1, participants completed a series of classical intertemporal choices in an individual and a joint decision-making condition. Here we aimed to reveal the possible benefit of social collaboration on discounting in classical intertemporal choices and to identify which mechanism underlies the expected dyadic variation. Based on these findings, we then conducted a second pre-registered study, in which the participants completed again the classical intertemporal choice task as well as the gamified delay discounting paradigm for which we had found the benefit of dyadic decision-making initially. In this study, we examined how the individual-collective-discrepancy and the interaction process between both participants were affected by the paradigm used and thus by different types of choices.

## Experiment 1

In Experiment 1, we aimed to generalize our findings from our former gamified discounting approach to classical discounting choices. Therefore, participants performed a classical delay discounting paradigm. Based on our former findings, that support the idea of less impulsive decision-making as a result of a social context as well as the literature that suggests a general beneficial effect of collaborative decision-making, we here hypothesized that smaller dyadic discounting occurs due to increased inhibition of impulsive responses. Therefore, we hypothesized that dyads show reduced discounting compared to their individual decision-making. We further tested whether this resulted from a general influence of the social context or from interactive processes between both participants.

### Methods

#### Ethics statement

The study was performed in accordance with the guidelines of the Declaration of Helsinki and of the German Psychological Society. An ethical approval was not required since the study did not involve any risk or discomfort for the participants. All participants were informed about the purpose and the procedure of the study and gave written informed consent prior to the experiment. All data were analysed anonymously. Participants received 5€ or partial course credit for their participation.

#### Participants

Sixty students of the Technische Universität Dresden, Dresden, Germany (39 females, *mean age* = 21.63, *SD* = 3.88), participated in the experiment. All participants had normal or corrected-to-normal vision. Each group consisted of two participants who were grouped based on their time slot preference yielding 30 random two-person groups (14 female-female; 5 male-male; 11 female-male). The participants did not know each other before the experiment. In order to ensure comparability, we used the same sample size of *n* = 30 dyads as in Schwenke et al. (2017).

The complete data set was collected incrementally on the following terms: Participants were excluded if their discounting in the individual condition was either too strong or too weak to prevent ceiling or floor effects on the individual condition. This should allow any modulation of the participant’s choice behaviour due to the experimental manipulation but also ensure that we do not produce any artificial effect due to a regression to the mean. To this end, we excluded participants with a relative frequency of sooner smaller (SS) choices over 80% (5 participants were excluded) and with a relative frequency of SS choices smaller than 20% (5 participants were excluded) in their individual decision-making condition. A total of 8 pairs were excluded. Data collection was stopped after the appropriate sample size of valid data sets was reached.

#### Procedure

Both participants were seated in front of two computer monitors on opposite sides of the laboratory, with the backs to each other. They were instructed to keep their eyes focused on their own screen and omit any communication with each other, verbally or nonverbally.

Their task was to execute a sequence of theoretical choices between a sooner but smaller (SS) or a later but larger (LL) choice option, e.g. 5€ in 1 day or 10€ in 5 days in an individual decision-making condition and in a dyadic decision-making condition. Bevor each condition started, the participants were instructed on the selection procedure through a standardized tutorial. In the dyadic condition, clear instructions were provided that they should imagine that each participant would get the, e.g. 5€ or 10€, so the decisions in the individual condition and in the dyadic condition per person were comparable.

**Fig. 1:**
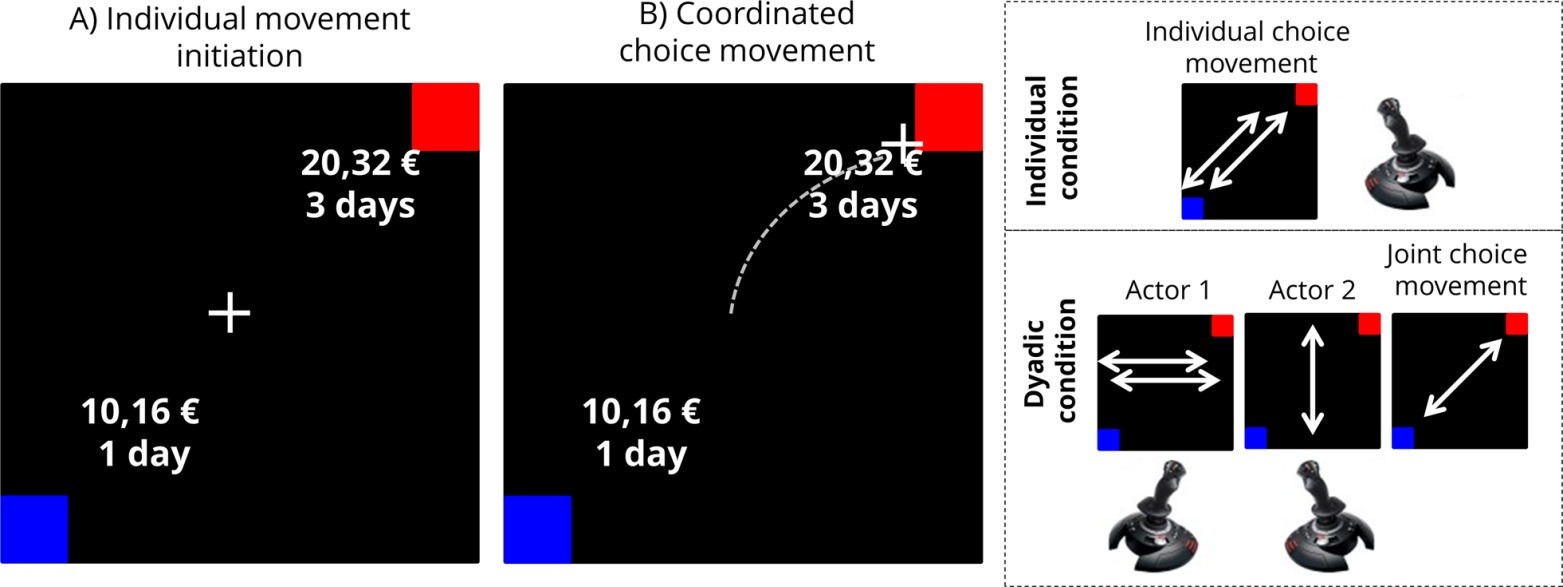
**Sketch of the experimental screen and procedure.** Left: each trial started with the presentation of the value in euros and the delay in days, and the cursor was locked in the centre of the screen. To select an option, the participants navigated the cursor into the colour-coded response box in the upper right/ lower left corner. Right: in the individual condition, each participant could reach their favoured response box by executing a diagonal joystick movement. In the dyadic condition, movement directions were split up to one participant controlling the vertical movement and the other controlling the horizontal movement while ignoring the other dimension. Because both cursor movements were added, the participants were able to move the jointly controlled cursor freely on the screen, comparable to the diagonal joystick movement in the individual condition

The choice selection procedure was comparable to our previous computer mouse based studies on individual discounting (Dshemuchadse et al., 2013). Each trial started with the presentation of one of the two choice options in the upper/right and the other in the lower/left square of the screen (see Fig. 1 left). The option value was presented in euros and the delay in days. Participants performed this task in two consecutive conditions (order of condition was randomized across dyads). In the individual condition, participants decided between both options by placing their cursor via diagonal joystick movement in an option associated target box in the upper/right or lower/left corner of the screen. In the dyadic condition, both participants had to coordinate their joystick movement which was split up to a horizontal and a vertical dimension (see Fig. 1 right).

The jointly controlled cursor only started to move after both participants had crossed an initial threshold of 80% of the maximum possible deflection of their joystick. After the cursor movement was initiated in this way, all amounts of change in joystick movement were effective. To end a trial, participants hat to coordinate their movements in order to make a final decision. In the case of conflict, the added cursor movement would move towards the top/left or bottom/ right segment of the screen in which no target box was located. To end the trial and perform a choice, both participants had to reach mutual consent, only interacting via jointly regulated cursor movement.

We do not set a time limit for decision-making. Between trials, the cursor was locked in the centre of the screen for the 0.3 s inter-trial interval (ITI). The next trial was started after the ITI when participants had relocated their joysticks in the centre position. This prevented a new decision movement from being accidently initiated before the new options were presented.

By following this procedure, we were able to distinguish three separate levels of decision: (1) the individual decision, calculated as the average of both individual decisions within the individual decision-making condition; (2) the pre-decision, calculated as the average of both individual decisions within the dyadic decision-making condition by measuring their initial individual joystick-movements; (3) the dyadic decision, calculated as the final decision of the participants through the unanimous assent of both.

#### Design

For both conditions, we generated trials according to the following scheme: We varied the value of the LL option (19.68€, 20.32€); the value of the SS option as a percentage of the value of the LL option (20%, 50%, 70%, 80%, 88%, 93%, 96,5%, and 99%); the delay of the SS option (0 and 7 days) and the interval between both options (1, 2, 3, 5, 7, 10, and 14 days). In each condition (individual, dyadic), participants completed 224 trials.

The order of the condition (individual first, joint first) was counterbalanced across all dyads. The position of the SS option (top/right vs. bottom/left segment) was constant throughout the experiment, but was counterbalanced across all dyads.

#### Data analysis

As dependent variables, we calculated the relative frequency of SS choices for all three levels of decision-making. Further, we measured the percentage of SS choices made in trials that showed initial conflict. All data and analyses for this study are openly available and can be downloaded from the Open Science Framework at osf.io/zq8y2. Data processing was carried out in MATLAB R2015a, and statistical testing was carried out using JASP 0.9.2. (JASP Team, 2019).

### Results

We first present the analyses of the extent of discounting by comparing the three levels of decision-making (individual decision, pre-decision, dyadic decision). Second, we present the analyses of choices in conflict trials, and third, we present analyses of the *order of condition*.

To avoid inflating statistical power, all measures for the individual decision and pre-decision were first aggregated for each individual participant and second averaged over both co-actors. All statistical results were Greenhouse–Geisser corrected where applicable, as will be indicated by a *.

For additional analyses on the log *k*-values, see Additional file 2, including results and correlations between both measures and the three levels of decision-making. The results of the analyses based on the log k-values and the analyses based on the SS choices essentially lead to the same results. All results of the log k-value analyses that lead to different results are explicitly stated in the following sections. We also evaluated the main analyses of interest with respect to the question how the results change when participants excluded due to very strong or weak discounting are gradually added to the analyses (see Additional file 3). Due to ceiling or floor effect, we no longer found any significant results after including the previously excluded pairs of participants.

#### Discounting

**Fig. 2:**
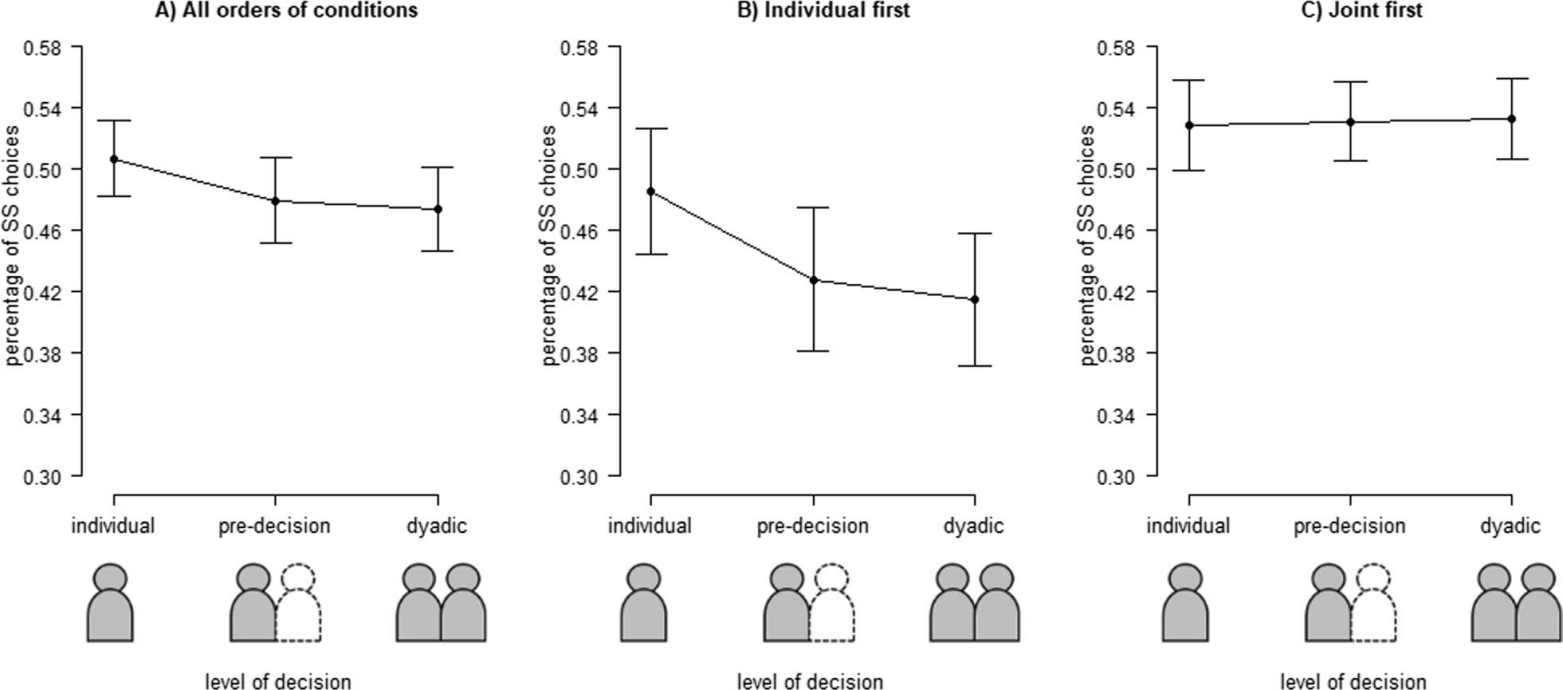
Results of Experiment 1. Relative frequency of SS choices in different levels of decision-making (individual decision, pre-decision, dyadic decision). Error bars indicate standard errors of the mean. The results are presented (**A**) without separation by the orders of conditions, (**B**) separately for the condition individual first, and (**C**) separately for the condition joint first

As a measure of discounting, we first calculated the relative frequency of choosing the sooner but smaller (SS) instead of the later larger (LL) option for all levels of decision-making. The descriptive statistics are presented in Table 1. To check whether individual and dyadic decision-making differed, we performed a repeated measures analysis of variance (ANOVA) on relative frequency of SS choices with the factor *level of decision* (individual decision, pre-decision, dyadic decision), yielding a significant main effect, *F*(1.268, 36.786) = 6.12, *p* = 0.013, *ηp*^*2*^ = 0.17*. Pairwise comparisons revealed that participants’ individual decisions resulted significantly more often in a SS choice compared to the pre-decisions, *t*(29) = 2.32, *p* = 0.028, *d* = 0.42, and the dyadic decisions, *t*(29) = 2.78, *p* = 0.009, *d* = 0.50. We found no significant difference between the pre-decision and the final dyadic decision,, *t*(29) = 1.12, *p* = 0.273 (see Fig. 2A).

**Table 1:** Descriptive statistics: percentage of SS choices in %

	All orders of conditions		Individual first		Joint first	
	M	SD	M	SD	M	SD
Individual	50.68	13.75	48.54	15.91	52.83	11.34
Pre-decision	47.94	15.22	42.81	17.99	53.06	9.98
Dyadic	47.38	14.79	41.49	16.59	53.27	10.18

#### Conflict

We next focused on the specific case of conflict trials, which we operationalized as an initially opposed joystick movement, i.e. one player attempted to move to the SS option while the other one attempted to move to the LL option. Overall, 16.44% (*SD* = 5.46%) of all choices were marked as trials with initially opposed preferences. We performed a one-sample *t*-test against 50% on the relative frequency of SS choices in conflict trials (*M* = 48.98%, *SD* = 16.01%) but found no significant effect, *t*(29) = 0.35, *p* = 0.73, indicating that trials with initial conflict did not result in more SS than LL choices.

#### Influence order of conditions

We checked for an influence of the *order of condition* (individual first, joint first). The descriptive statistics of the percentage of SS choices in all levels of decision-making are shown also separately by order of condition in Table 1. We performed a repeated measures analysis of variance (ANOVA) on relative frequency of SS choices with the factors *level of decision* (individual decision, pre-decision, dyadic decision) and *order of condition* (individual first, joint first), yielding a significant main effect of *level of decision-making*, *F*(1.33, 37.17) = 8.04, *p* = 0.004, *ηp*^*2*^ = 0.22*, and an interaction effect between the *level of decision-making* and the *order of condition, F*(1.33, 37.17) = 10.10, *p* = 0.001, *ηp*^*2*^ = 0.27^*^. We found no significant main effect of *order of condition*, *F*(1, 28) = 3.05, *p* = 0.092. For post hoc comparison we conducted paired *t*-tests for each *order of decision-making* separately. When the individual decision-making condition was conducted first, participants showed higher discounting in the individual decision compared to the pre-decision, *t*(14) = 4.48, *p* < 0.001, *d* = 1.26, and the dyadic decision, *t*(14) = 7.07, *p* < 0.001, *d* = 1.83. In the comparison of the pre-decision and the dyadic decision, there is a difference between the analysis of the SS choices and the log *k*-values. The pre-decision and dyadic decision did not significantly differ when analysing the SS choices, *t*(14) = 1.49, *p* = 0.159, while the log *k*-values showed a significant difference, *t*(14) = 2.25, *p* = 0.041, *d* = 0.58 (see Fig. 2B). We found no significant differences between the three levels of decision-making for the participants who conducted the dyadic decision-making condition first, all *t* < 0.54 and all *p* > 0.600 (see Fig. 2C).

### Discussion

In Experiment 1, we aimed to generalize our findings from our former gamified discounting approach to classical discounting choices. Based on our former findings, we hypothesized that participants in a joint decision-making condition would show reduced discounting compared to an individual condition. When we traced three levels of decision-making (the individual decision in the individual condition, the pre-decision as the very first indication of individual preferences within the dyadic condition, and the final dyadic condition in the joint decision-making condition), we further hypothesized that this reduced discounting would occur as a consequence of dyadic collaboration rather than the social context itself.

Our findings demonstrated some evidence that there is less discounting, i.e. a lower relative frequency of sooner but smaller (SS) choices in the final dyadic condition compared to the individual condition. Although this finding is consistent with our former study, two important differences came apparent. First, this pattern only applied to participants who conducted the individual condition first, while no significant differences between all three levels of decision-making were found for participants who conducted the dyadic condition first. Second, a crucial difference laid in the process by which this differences occurred. For pairs that performed the individual condition first, we found significant difference in the percentage of SS choices between the individual decision and the pre-decision but no significant difference between the pre-decision and the final dyadic decision. This indicates that, in contrast with our previous study, the social context itself rather than social collaboration leads to lower dyadic discounting. In additional analyses, we also found substantial evidence that the difference between individual decision and pre-decision is caused purely by the social context, since in this level of decision-making no adjustment to the identified option preference of the partner takes place (for details see supplementary material S4). It is also important to note that we did not find an influential effect of the order of condition in our former study (see Additional file 1 for a subsequent analysis).

The question arises why the findings contradict our former research. One could assume that this modulation resulted from the methodical distinctiveness of the both paradigms itself. In the gamified paradigm, we used a real-time reference in which the choices were experienced by the participants in terms of an actual period of waiting and a monetary pay out. Also, we were able to objectively classify each choice option in either being the optimal or the non-optimal option. Here in contrast, classical delay discounting choices were presented as hypothetical choices with relatively large delays and values, which only allowed the measurement of the subjective devaluation instead of an additional measurement of efficiency.

However, in both studies, our observations were restricted to one type of discounting paradigm, a classical approach, or a gamified approach of delay discounting, leaving it unclear whether the option presentation determines the process of joint delay discounting in such a substantial way. For that reason, we aimed to study this possible difference and therefore performed Experiment 2, in which we systematically compared the option presentation of both paradigms and their implications on the joint delay discounting.

## Experiment 2

Experiment 2 was a preregistered study (osf.io/rjuf3). Participants performed both paradigms, the classical delay discounting and the gamified delay discounting paradigm, in two sessions with an interval of at least 7 days. Based on our previous findings, we hypothesized (1) that in both paradigms participants would show reduced discounting in the dyadic condition compared to their individual discounting behaviour but (2) that the mechanisms underlying this variation depend on the option presentation and thus the paradigm. According to our results in Experiment 1, we expected that in case of classical option presentation the social context itself determines the dyadic decision. In consequence to the results in our previous study (Schwenke et al., 2017), we expected that in case of using a real-time reference inner group interchange determines the dyadic decision. Additionally, we hypothesize that trial with initial conflict will result more often in LL choices but only in the paradigm using a real time reference. To check whether participants’ discounting was stable over the 1-week period, we determined the participants’ *k*-value using the well-established monetary choice questionnaire (Kirby et al., 1999) and hypothesized (3) a high level of stability between both sessions. If participants showed highly stable discounting, their modified decision-making (between experimental conditions) would be due to experimental manipulation rather than general instability of their discounting.

### Methods

#### Ethics statement

The study was performed in accordance with the guidelines of the Declaration of Helsinki and of the German Psychological Society. An ethical approval was not required since the study did not involve any risk or discomfort for the participants. All participants were informed about the purpose and the procedure of the study and gave written informed consent prior to the experiment. All data were analysed anonymously. Participants received 15€ or partial course credit as well as the money they collect within the gamified delay discounting task for their participation. They could collect 2–4 euros, but the amount earned was rounded up so that all participants received an identical bonus payment (in the preregistration this was explained in an unclear way).

#### Participants

For data analysis, we included data of sixty students of the Technische Universität Dresden, Dresden, Germany (42 females, *mean age* = 27.12, *SD* = 10.07). All participants had normal or corrected-to-normal vision. Each group consisted of two participants who were grouped based on their time slot preference yielding 30 two-person groups (12 female–female; 1 male–male; 17 female–male). The participants did not know each other before the experiment.

In order to ensure comparability, we used the same sample size of *n* = 30 dyads as in Experiment 1 and the original gamified study (Schwenke et al., 2017). Again, we excluded the data from participants who showed very high or low discounting (percentage of SS choices lower than 20% or higher than 80%) in any of the two paradigms (classical paradigm: 6 participants were excluded due to too low discounting, 1 participant was excluded due to too high discounting; gamified: 3 participants were excluded due to too low discounting; in total, we excluded 9 pairs from the experiment). Data collection was stopped after the appropriate sample was size was reached.

#### General procedure

The study took place in two separate sessions with an interval of at least 7 days, the participants collaborated with the same person in both sessions. In each session, the participants completed one paradigm, the classical delay discounting or the gamified delay discounting paradigm in an individual decision-making condition and a dyadic decision-making condition. They were seated in front of two computer monitors on opposite sides of the laboratory, with the backs to each other. They were instructed to keep their eyes focused on their own screen and omit any communication with each other, verbally or nonverbally, during the experiment and after session 1. After the experimental task, they completed the monetary choice questionnaire in each session (Kirby et al., 1999).

#### Task procedure

##### Classical intertemporal choice

The classical paradigm was carried out as described in Experiment 1.

##### Gamified intertemporal choice

Participants again were asked to performed a series of SS versus LL choices.

**Fig. 3:**
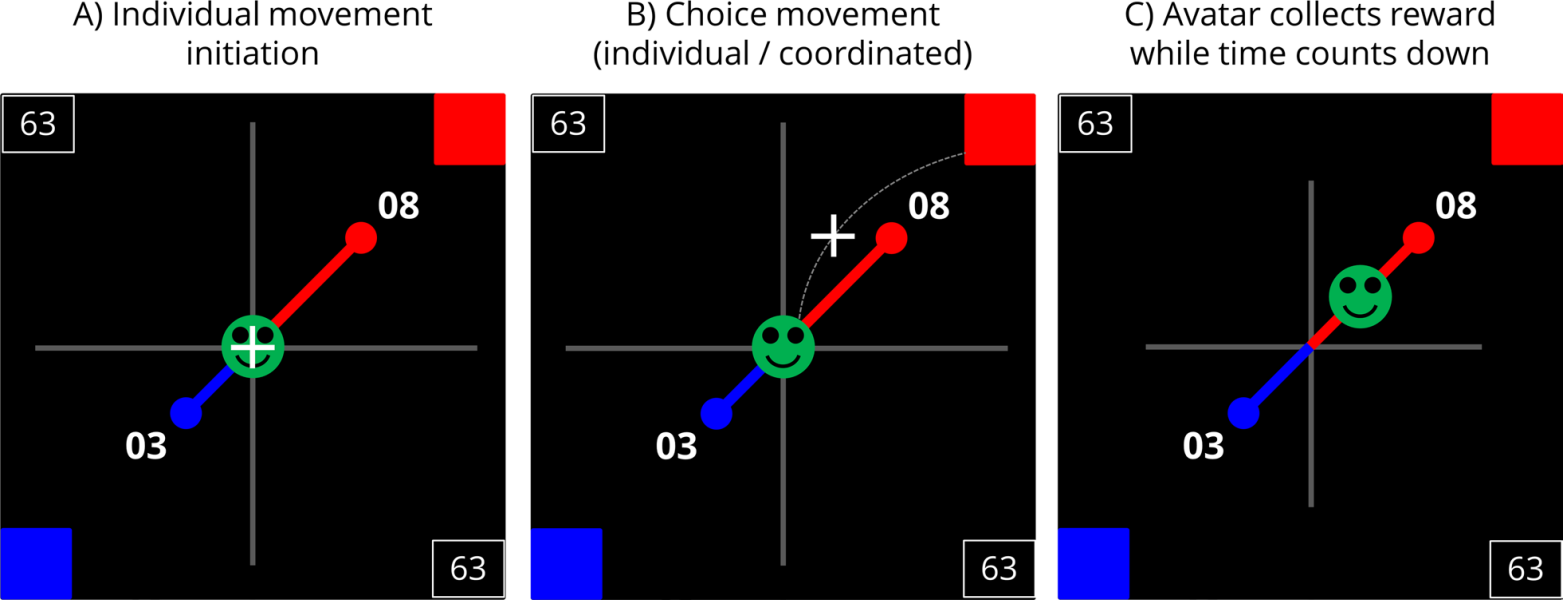
Sketch of the experimental screen and procedure. (**A**) Each trial started with the presentation of the avatar and the two decision options. The cursor was locked in the centre of the screen. The current score that the participants had already collected was shown in the top/left and the bottom/right corner of the playing area. (**B**) To select an option, participants navigated the cursor into the colour-coded response box in the upper right/ lower left corner of the playing area. (**C**) After reaching a target box, the avatar started to move along a conjunction line from the centre of the screen to the place where the chosen option was presented. While the avatar was moving, a limited amount of time was counted down (demonstrated by reducing the length of the grey crossed lines in the background). Due to the greater distance to the avatar, more time was required to collect the later/larger reward

The gamified paradigm followed the procedure comparable to our former approach (Schwenke et al., 2017) in which we presented a SS and a LL option in real time: Each trial started with the presentation of an avatar in the centre of a computer screen and the two decision options, one at the upper/right and the other at the lower/left square of the screen. Each option’s value was displayed using numbers which were connected to the avatar by a diagonal line. The length of these lines indicated the temporal delay of the options (see Fig. 3A). The joystick-based choice procedure was identical to Experiment 1. After reaching a response box with the cursor, the avatar started to move automatically along the line to the chosen option while a limited amount of the collecting-time was counted down (i.e. 66 s within one block) which was demonstrated by reducing the length of the grey crossed lines in the background. This countdown was paused when the avatar reached the chosen option and the next trial was started. According to the option positions and distance to the avatar, the avatar needed relatively more time to gain the LL option than the SS option (see Fig. 3C).

Comparable to Experiment 1, we were able to distinguish three separate levels of decision: (1) the individual decision; (2) the pre-decision; (3) the dyadic decision.

#### Design

In the classical paradigm, we used the same trials as in Experiment 1. For the gamified paradigm, we generated trials according to the following scheme: The values of the options ranged whole-numbered from 01 to 05 credits for the SS option and from 06 to 10 credits for the LL option, whereby the values of the SS and the LL option together always resulted in 11 points (small/large pairs were: 1/10, 2/9, 3/8, 4/7, 5/6). The SS option could be reached in 1, 3 and 6 units of time, whereas the LL option could be reached in 2, 3, 4, 5, 7, 8, 9, 12, 14 and 17 units of time (sooner/later pairs were: 1/2, 1/3, 1/7, 1/12, 3/4, 3/5, 3/9, 3/14, 6/7, 6/8, 6/12, 6/17, resulting in minimal interval of 1 unit and a maximal interval of 11 units of time). This resulted in a pool of 60 possible types of value-delay combinations. Participants performed 4 blocks in both conditions, each lasting 66 s collection time (i.e. time available for the avatar to move to the chosen options).

#### Data analysis

We measured the relative frequency of SS choices for all three levels of decision-making and computed the difference between *individual decision and pre-decision* and between *pre-decision and dyadic decision*. Further, we measured the percentage of SS choices made in trials that showed initial conflict. The temporal stability of delay discounting was assessed by measuring the *k*-value with the monetary choice questionnaire (Kirby et al., 1999). Data processing was carried out in MATLAB R2015a and statistical testing was carried out using JASP 0.9.2. (JASP Team, 2019).

The whole study followed a 2 (*paradigm*) × 3 (*level of decision-making*) within-subjects design. Participants went through the whole design within two sessions. The order of paradigm (classical intertemporal choice first, gamified intertemporal choice) was counterbalanced across participants. Similar, the order of condition (individual first, joint first) was counterbalanced across participants but was held constant across paradigms/ sessions for each participant/ dyad. The position of the SS option (upper/right, lower/left) was counterbalanced across participants, but was held constant across conditions and paradigms.

### Results

First, we present the confirmatory analyses and, second, the exploratory analyses to examine the influence of the experimental design on the results. To avoid inflating statistical power, all measurements of the individual decision and the pre-decision were aggregated for each individual participant and further averaged over both co-actors in order. All statistical results were Greenhouse–Geisser corrected when applicable, as will be indicated by a *. Similar to experiment 1, all analyses were also conducted on the log *k*-value (see Additional file 2 for results and correlations between both measurements and the three levels of decision-making). The results of the analyses based on the log k-values and the analyses based on the SS choices essentially led to the same results. All results of the log k-value analyses that lead to different results are explicitly stated in the following sections. We also evaluated the main analyses of interest with respect to the question how the results change when participants excluded due to very strong or weak discounting are gradually added to the analyses (see Additional file 3). We did not identify any changes in the significance of the results.

#### Confirmatory analyses

**Fig. 4:**
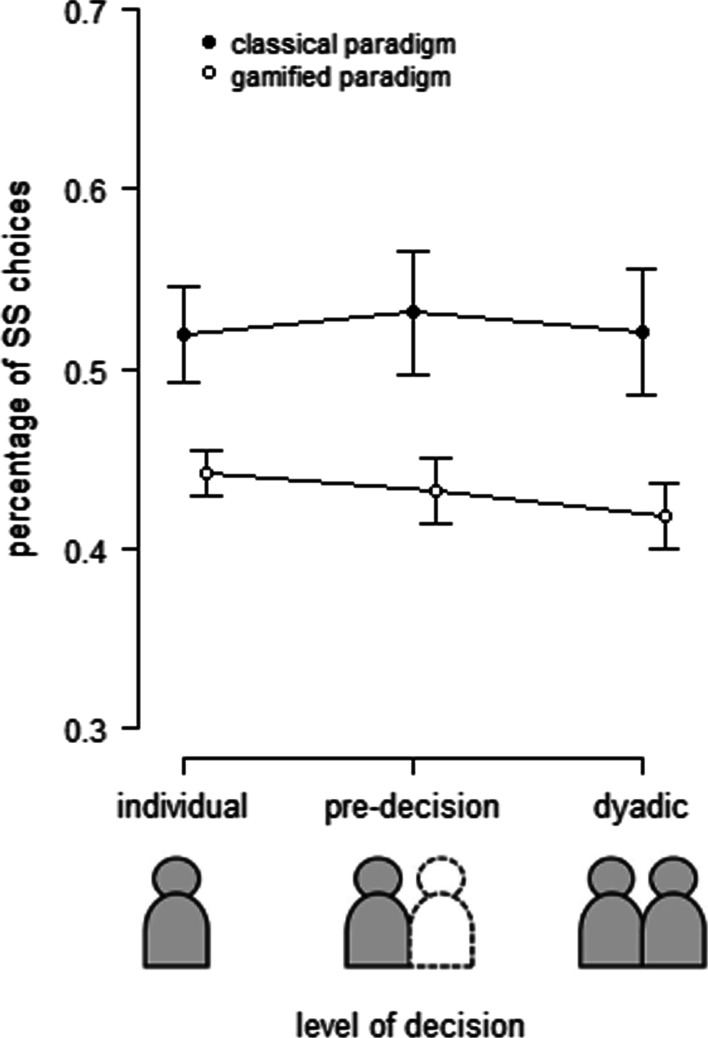
Results of confirmatory analyses of Experiment 2. Relative frequency of SS choices in different levels of decision-making (individual decision, pre-decision, dyadic decision). Error bars indicate standard errors of the mean

Hypothesis 1 stated that participants in the dyadic decision-making condition discount less compared to the individual decision-making condition. To test this hypothesis, we performed a repeated measures analysis of variance (ANOVA) with the factor *paradigm* (classical intertemporal choice, gamified intertemporal choice) and *condition* (individual condition, dyadic condition) on the percentage of SS choices. The descriptive statistics are presented in Table 2. We found a significant main effect for the factor *paradigm*, *F*(1, 29) = 8.03, *p* = 0.008, *ηp*^*2*^ = 0.22, indicating that participants chose the SS option in the gamified paradigm less often. Further, we found no significant effect for *condition*, *F*(1, 29) = 2.74, *p* = 0.109, or for the interaction between *paradigm* and *condition*, *F*(1, 29) = 0.73, *p* = 0.402 (see Fig. 4). Concluding these findings, there are no indications to support our hypothesis.

**Table 2:** Descriptive Statistics: percentage of SS choices in %

	Classical		Gamified	
	M	SD	M	SD
Individual	51.91	14.45	44.21	7.19
Pre-decision	53.11	18.61	43.25	10.04
Dyadic	52.04	19.07	41.79	9.82

Hypothesis 2 stated that the social context itself would affect the dyadic decision-making in the classical paradigm, while inner group interchange would affect the dyadic decision-making in the gamified paradigm. Following this reasoning, the individual choice should differ from the pre-decision in the classical paradigm, while the pre-decision should differ from the dyadic decision in the gamified paradigm. Further, in conflict trials with initially opposing preferences, the LL option should be selected in more than 50% of all trials. To examine these assumptions, we performed a repeated measures ANOVA with the factors *paradigm* (classical, gamified) and *differences between levels of decision-making* (difference between individual decision and pre-decision, difference between pre-decision and dyadic decision) on the percentage of SS choices (see Table 2 and Fig. 4). We found no significant effect, all *F*(1, 29) < 2.50, all *p* > 0.125. Second, we performed a one-sample *t*-test against 50% on the percentage of SS choices in conflict trials for each paradigm separately. We found significant effects for both classical delay discounting (*M* = 44.02, *SD* = 12.77), *t*(29) = 2.29, *p* = 0.030, *d* = 0.42, and gamified delay discounting (*M* = 43.96., *SD* = 9.68), *t*(29) = 3.10, *p* = 0.004, *d* = 0.56, indicating that in conflict trials the SS option was chosen less often.

In summary, we found no indication for any differential impact of the type of paradigm on dyadic delay discounting and thus no indication to support hypothesis 2.

We further hypothesized a high stability of discounting over a 1-week period. We therefore determined participants’ *k*-value via the monetary choice questionnaire (Kirby et al., 1999) and computed the Spearman correlation coefficient between both sessions, which indicated a high level of stability, *Rho* = 0.722, *p* < 0.001.

#### Exploratory analyses

In light of the results of Experiment 1, we next checked whether the experimental design may have affected the difference between individual and dyadic decision-making. First, we performed a repeated a measures analyses (ANOVA) with the within-factors *paradigm* (classical, gamified) and *level of decision-making* (individual, pre-decision, dyadic decision) and the between factor *order of condition* (individual first, joint first) on the relative frequency of SS choices and found a significant main effect for *paradigm, F*(1, 28) = 6.64, *p* = 0.016, *ηp*^*2*^ = 0.19. Further, we found significant interaction effects between *paradigm* x *order of condition*, *F*(1, 28) = 4.94, *p* = 0.034, *ηp*^*2*^ = 0.15, *level of decision-making* x *order of condition, F*(1.23, 34.31) = 6.25, *p* = 0.013, *ηp*^*2*^ = 0.18*, and the interaction between *paradigm* x *level of decision-making* x *order of condition*, *F*(1.20, 33.75) = 5.65, *p* = 0.018, *ηp*^*2*^ = 0.17*. We found no significant main effects of *level of decision-making*, *F*(1.23, 34.31) = 2.43, *p* = 0.123, or *order of condition*, *F*(1, 28) = 1.22, *p* = 0.279*.* There was also no significant interaction effect between *paradigm x level of decision-making*, *F*(1.20, 33.75) = 1.49, *p* = 0.236.

Second, we conducted a similar analysis with the within-factors *paradigm* (classical, gamified) and *level of decision-making* (individual, pre-decision, dyadic decision) and the between factor *order of paradigm* (classical first, gamified first). Except for a significant main effect of *paradigm*, *F*(1, 28) = 6.13, *p* = 0.020, *np*^*2*^ = 0.18, we found no other significant main or interaction effects, all *F* < 2.39 and all *p* > 0.133, indicating that the *order of paradigm* had no influence on the decision-making.

To get a deeper insight into the found effects of the factor *order of condition*, we performed post hoc comparisons in which we reduced the level of complexity stepwise, performing ANOVAs with the factors *level of decision-making* and *order of condition* similarly to Experiment 1, but separately for each paradigm, followed by *t*-tests for significant effects. The descriptive statistics of the percentage of SS choices are presented separately by paradigm and order conditions in Table 3.

**Table 3:** Descriptive statistics: percentage of SS choices in %

	Classical				Gamified			
	Individual first		Joint first		Individual first		Joint first	
	M	SD	M	SD	M	SD	M	SD
Individual	49.03	13.54	54.78	15.22	46.25	6.36	42.17	7.60
Pre-decision	46.14	19.91	60.08	14.74	45.52	8.88	40.97	10.90
Dyadic	44.77	20.94	59.31	14.19	44.34	9.18	39.25	10.07

##### Classical paradigm

**Fig. 5:**
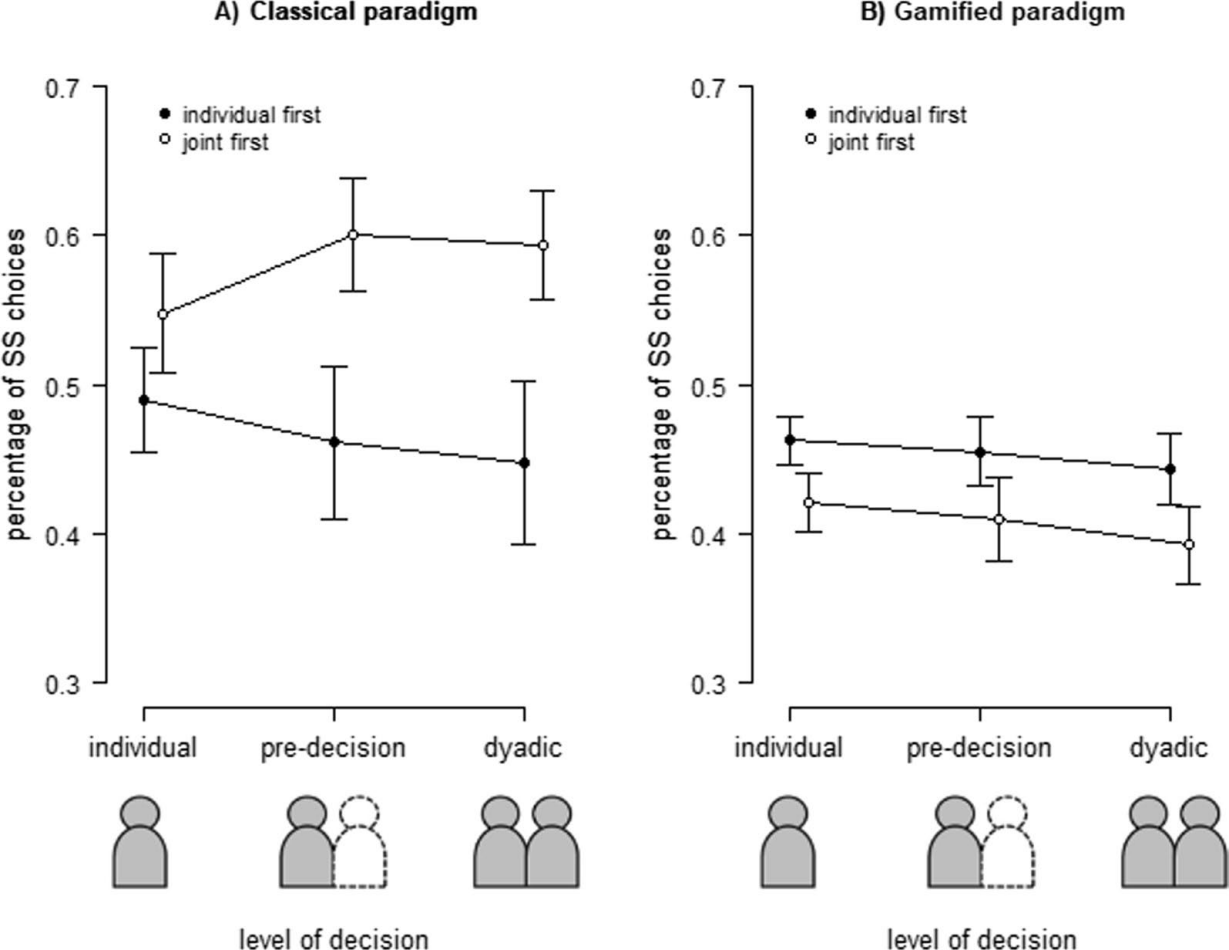
Results of exploratory analyses of Experiment 2. Relative frequency of SS choices in different levels of decision-making (individual decision, pre-decision, dyadic decision). Error bars indicate standard errors of the mean. (**A**) Results of the classical intertemporal choice paradigm separated by the order of conditions. (**B**) Results of the gamified intertemporal choice paradigm separated by the order of condition

In the repeated measures ANOVA with the factors *level of decision-making* and *order of condition*, we found no main effect, all *F* < 3.67 and all *p* > 0.065, but a significant interaction effect between both factors, *F*(1.20, 33.61) = 9.55, *p* = 0.003, *ηp*^*2*^ = 0.25*. To get a deeper understanding of this interaction, we conducted post hoc paired *t*-tests for each group separately, similar to experiment 1. We found that when the individual condition was conducted first, we found no differences between the levels of decision-making, all *t* < 1.90 and all *p* > 0.078. When participants performed the joint condition first, they showed lower discounting in the individual decision compared to the pre-decision, *t*(14) = 3.44, *p* = 0.004, *d* = 0.89. The comparison of the individual and the dyadic decision results in differences between the analysis of SS choices and log *k*-values. The participants showed lower discounting in the individual compared to the dyadic decision when analysing the SS choices, *t*(14) = 3.02, *p* = 0.009, *d* = 0.78, while the log *k*-values showed no significant difference, *t*(14) = 1.948, *p* = 0.072. The pre-decision and dyadic decision did not significantly differ, *t*(14) = 1.40, *p* = 0.184 (see Fig. 5A).

##### Gamified paradigm

We conducted the same analyses and found a significant main effect for *level of decision-making F*(1.23, 34.46) = 3.66, *p* = 0.021, *ηp*^*2*^ = 0.12* but no main effect for *order of condition* and no interaction between both factors, all *F* < 2.18 and all *p* > 0.151. Because of the lack of an interaction effect, we conducted post hoc pairwise *t*-tests between all three levels and found that participants’ dyadic decision resulted significantly less often in a SS choice compared to the individual decision, *t*(29) = 2.40, *p* = 0.023, *d* = 0.44. The difference between individual decision and pre-decision did not reach significance, *t*(29) = 0.90, *p* = 0.379. The paired t-tests comparing the pre-decision and dyadic decision resulted in differences for the analyses of SS choices and log *k*-values. The analysis of SS choices showed a significant difference between the pre-decision and the dyadic decision, *t*(29) = 3.50, *p* = 0.002, *d* = 0.64, while the analysis of log *k*-values did not lead to a significant difference, *t*(29) = 1.11, *p* = 0.275 (see Fig. 5B).

### Discussion

In Experiment 2, we systematically tested for any experimental influences of the option presentation on the difference between individual and joint delay discounting. Participants performed two versions of discounting tasks, classical intertemporal choices and gamified delay discounting choices, in two sessions with an interval of at least 7 days.

We found no evidence to support our preregistered hypotheses. Thus, we could not prove a paradigm-independent decrease in discounting in the dyadic condition compared to the individual condition, nor could we show with certainty that the mechanism underlying the individual-dyadic discounting discrepancy depends on the paradigm and thus the type of choices.

In Experiment 1, when the classical intertemporal choice paradigm was used, we found that the order of conditions affected the results. Thus, we examined whether the experimental design also had an influence on the results in Experiment 2. This is not the case for the order of paradigms but for the order of conditions. For this reason, we also conducted exploratory analyses on the influence of the order of conditions separately for both paradigms in Experiment 2. In the classical intertemporal choice paradigm, as in experiment 1, there was again an influence of the order of conditions. Participants who performed the individual condition first showed no differences between the levels of decision-making. Participants who performed the joint condition first showed lower discounting in the individual decision than in the pre-decision and the dyadic decision. Thus, the pattern of results differs slightly from Experiment 1. In the gamified intertemporal choice task, however, we found no influence of the order of conditions. In this paradigm, participants showed lower discounting in the dyadic decision than in the individual decision and pre-decision. This pattern is evidence for an influence of inner group interchange on dyadic decision-making in the gamified paradigm and is consistent with our results from (Schwenke et al., 2017).

## General discussion

Humans frequently perform a diversity of delay related decisions collaboratively in everyday life. With a few exceptions, however, the role of social influences on such intertemporal choices has largely been overlooked. In this study, we aimed to fill this gap and studied the effects of social collaboration on intertemporal choices. Starting from initial results in a gamified delay discounting paradigm in which we observed that discounting is lower for collaborative choices than for individual choices and identified that this decrease in discounting was caused by the interactive process of the participants, we asked whether these results are generalizable to classical intertemporal decisions and how the methodical differences between the gamified and classical intertemporal choice paradigm may affect the processes underlying dyadic discounting. We therefore conducted two experiments. In Experiment 1, we aimed to generalize our findings from our former gamified discounting approach (Schwenke et al., 2017) to classical discounting choices. Due to inconclusive results of this study, we then conducted a pre-registered experiment 2, in which both paradigms, the classical and the gamified version, were systematically compared in a within-subject design. To concisely frame the results of these two experiments, we will first briefly recap our initial research and then outline the findings of the new experiments.

In our initial study, we applied a novel gamified discounting task in which the participants decided between a sooner but smaller (SS) and a later but larger (LL) choice in an individual and a dyadic decision-making condition via a non-verbal, jointly controlled cursor movement coordination. Importantly, participants here collected the preferred rewards in real time on a trial-to-trial basis instead of choosing between hypothetical rewards in a distant future. Additionally, our paradigm provided the possibility to classify each choice as being an optimal or non-optimal decision according to a normative reference. We have traced the decision-making process by disentangling three levels of decision-making: the individual decision within an individual decision-making condition (individual decision), the pre-decision as the first individual indication of preference within the joint decision condition (pre-decision), and the final dyadic decision (dyadic decision). The participants showed reduced discounting in their final dyadic decision compared to their individual decision and their pre-decision indicating that the lower dyadic discounting resulted as a consequence of the interactive process itself rather than as a consequence of the influence of the mere presence of somebody else.

In Experiment 1, participants completed a similar choice selection procedure via non-verbal cursor movement coordination but performed classical, i.e. long-term and hypothetical intertemporal choices. We found some evidence replicating the main finding, such as that participants showed lower discounting in their joint decision-making compared to their individual decision-making. As regards to the underlying mechanism, however, we found that the social context itself played an important role in the difference between individual and joint intertemporal choices. Here, participants have already adapted their choices in their pre-decision indicating a high influence of the social context itself. Importantly, an exploratory analysis of the order of condition revealed that this pattern only applied to participants who conducted the individual condition first. However, we found no differences between the three levels of decision-making for participants who performed the joint condition first.

In the following pre-registered Experiment 2, we examined the expected discounting decrease in joint versus individual decision-making and systematically tested whether the differences in the underlying processes are due to the experimental specifics of the two paradigms. Participants performed both, the classical and the gamified version, in a within-subject design. Our findings, however, were inconclusive. The results showed no paradigm-independent decline in discounting in the joint compared to the individual decision-making. Analysing the results separately according to paradigms and the order of the conditions, a differentiated pattern was revealed. In the classical paradigm, the results were inconsistent with Experiment 1. The order of conditions affected the intertemporal decision-making, but in contrast to Experiment 1, we found no differences between the levels of decision-making for participants who processed the individual condition first. For participants who performed the joint condition first, we also found a different pattern of results than in Experiment 1. For the gamified paradigm, however, the order of condition played no role in the participants’ intertemporal decision-making and we found a lower frequency of SS choices in the dyadic condition which occurred as a consequence of the dyadic interactive coordination and not due to the influence of the presence of the co-actor. This pattern is consistent with and replicates the results in our former study (Schwenke et al., 2017).

What can we conclude from those findings? It appears that the general assumption that dyadic discounting is less impulsive than individual discounting is not supported by our findings (for similar conclusion see Bixter et al., 2017), but one could conclude that social influences play an important role in intertemporal decision-making. However, the specific implications are shaped by different aspects of the decision context itself. One important aspect seems to be the type of choices and thus the option presentation, as hypothesized in our study.

In contrast to classical intertemporal choices, the results of our gamified intertemporal choice paradigm reliable showed a reduction in discounting in the dyadic compared to the individual decisions which was caused by social collaboration and was not influenced by the order of conditions, i.e. whether the individual or the dyadic decision-making was conducted first. This may be caused by the following features of the gamified paradigm: First, the gamified version operates with a real-time experience of the delay of the preferred option in that participants have to wait until the avatar has collected the preferred option. Second, each decision has a monetary consequence for the payment of compensation at the end of the experiment. Thus, they are not hypothetical monetary gains. This could have led to a more systematic strategy resulting in higher decision consistency. The third feature is the objective optimality of one option in each trial. By implementing such a normative reference, the subjective devaluation can be separated from the objective efficiency of the decision. Exactly this normativity could have been the operating mechanism behind the stability of our findings, though it did not prevent the participants from choosing differently in the individual and the dyadic condition. Our paradigm of classical intertemporal decisions does not have these features. This could be a reason why the result, as Experiment 1 and 2 showed, are less stable and more susceptible by the context in which they are made. For example, the order of conditions played a notable but inconclusive role.

These findings contrast with what is often predicted by traditional intertemporal models, namely that there are stable tendencies to be impulsive or patient. Recent research started to question this stability by highlighting a number of different contextual influences (Lempert & Phelps, 2016): A shift in preference can be induced by e.g. framing, or the state of the decision maker. This reasoning is in line with other discounting studies investigating forms of social influences on discounting such as the observation of the choices of others, or surrogate decision-making (Calluso et al., 2017; Gilman et al., 2014; Tunney & Ziegler, 2015; Ziegler & Tunney, 2012).

### Limitations

The research question we studied in this work was whether the methodical differences between a classical and a gamified discounting paradigm can affect the processes underlying dyadic discounting. It is important to point out that the particular level of interaction which be focused on is the non-verbal interaction dynamic between two co-acting participants coordinating each other via joystick movements. Although this level of interaction appears to be very limited, interactional processes are situated on a variety of different low and high level behavioural patterns, for instance dialog, gesture (Maricchiolo et al., 2011), eye-movement (Peshkovskaya et al., 2017), and also on more conceptual levels, e.g. shared cognition (Cooke et al., 2013) or synergy (Fusaroli & Tylén, 2016). Research on such high-level open-ended interaction dynamics thereby often lacks an experimental structure with sufficient experimental control. Here we have striven for a minimalistic but highly constrained approach in order to break down the interaction sequence into separately analysable steps. Distinguishing the three levels of decision-making allowed a novel insight into the underlying mechanisms of delay discounting. Focussing on this elementary level, we do not claim to study the phenomenon of interactional processes to the full extent. Instead, we aimed to provide a novel approach to tracing non-verbal communication as one possible approach among many others (Abney et al., 2014). Another important limitation concerns the effect of the order of decision-making. The observed effects were based only on a very small sample size (i.e. only 15 dyads performed the individual condition first and 15 dyads performed the joint condition first) which does not allow a conclusive interpretation. Although this research indicates that the classical choices were more likely to be influenced by contextual factors and therefore did not provide reliable results, further research with a bigger sample size would be necessary to provide more insight into the specific effects and underlying mechanisms.

### Conclusion

Our results indicate that the social influences are of considerable importance for intertemporal decision-making. In our study, we primarily examined the impact of two versions of option presentation, a classical and a gamified version, on the difference between individual and dyadic discounting. We found that dyadic discounting is not less impulsive than individual discounting regardless of circumstances. In a gamified intertemporal choice paradigm with a normative reference and real consequences of each decision, dyadic discounting is lower than individual discounting and this reduction is likely caused by social collaboration. However, classical intertemporal choices are considerably influenced by the context. Although our results did not support our initial hypotheses to the full extent, this research revealed surprising insights into the fragility and suggestibility of social discounting choices. In view of the present work, we conclude that the interaction of social aspects and contextual factors of delay discounting is an important subject for future research.

## Supplementary Information

**Additional file 1:** Influence of the order of condition (individual first vs joint first) on the discounting behavior in our previous study (Schwenke et al., 2017).

**Additional file 2:** Results of our study based on log *k*-values as measure of discounting.

**Additional file 3** Results of the main analyses with gradual inclusion of participants previously excluded due to very strong or weak discounting.

**Additional file 4:** Analysis of the factors influencing the participants' behavior in the pre-decision.

## Data Availability

Primary data and analysis scripts (MATLAB) are publicly available at the Open Science Framework: osf.io/zq8y2. Experiment 2 was pre-registered at the Open Science Framework: osf.io/rjuf3. We report achieved power for the used sample size, all data exclusions, and all relevant measures and manipulations in the study.
