# Supplementary material for: Effects of individual and dyadic decision-making and normative reference on delay discounting decisions
Source: Cogn Res Princ Implic. 2022 Jul 28;7:71. doi: 10.1186/s41235-022-00422-5 (PMC9334506; doi:10.1186/s41235-022-00422-5)
Supplement: Supplementary file 1 — Additional file 1: Influence of the order of condition (individual first vs joint first) on the discounting behavior in our previous study (Schwenke et al., 2017). [file 41235_2022_422_MOESM1_ESM.docx]

**Effects of individual and dyadic decision making and normative reference on delay discounting decisions**

Supplement Materials S1

# Diana Schwenke, Peggy Wehner, Stefan Scherbaum

# Department of Psychology, Technische Universität Dresden, Dresden, Germany

We performed a subsequent analysis on the data of our former study, publicly available at [osf.io/rjuf3](https://osf.io/rjuf3).

We conducted a repeated measures analysis of variance (ANOVA) with the within-factor *level of decision making* (individual, pre-decision, dyadic decision) and the between-factor *order of condition* (individual first, dyadic first) on the measured frequency of SS choices.

We found no significant main or interaction effect relating to the order of decision-making, all *F* < 1.726, *p* > .200.
