# Supplementary material for: Effects of individual and dyadic decision-making and normative reference on delay discounting decisions
Source: Cogn Res Princ Implic. 2022 Jul 28;7:71. doi: 10.1186/s41235-022-00422-5 (PMC9334506; doi:10.1186/s41235-022-00422-5)
Supplement: Supplementary file 2 — Additional file 2: Results of our study based on log k-values as measure of discounting. [file 41235_2022_422_MOESM2_ESM.docx]

**Effects of individual and dyadic decision making and normative reference on delay discounting decisions**

Supplement Materials S2

# Diana Schwenke, Peggy Wehner, Stefan Scherbaum

# Department of Psychology, Technische Universität Dresden, Dresden, Germany

In addition to the analysis of the relative frequency of the sooner but smaller (SS) choices, we additionally calculated the logarithmic *k*-value of the hyperbolic function estimated according to Kirby et al. (1999)^[[1]](#footnote-1)^ for each level of decision-making:

V = A / (1+ k*D), where V is the value of the delayed reward A at the delay D.

Here, an increased *k*-value means a higher extent of discounting. All statistical results were Greenhouse-Geisser corrected where applicable*.

**Experiment 1**

We performed a repeated measures analysis of variance (ANOVA) on the log *k*-values with the factor *level of decision* (individual decision, pre-decision, dyadic decision), yielding a significant main effect, *F*(1.287, 37.328) = 12.40, *p*< .001, *ηp^2^*= 0.30^*^. Post hoc comparison revealed significantly lower discounting in the final dyadic decision compared to the individual decision, *t*(29) = 3.80, *p*< .001, *d*= 0.69. We further found lower discounting in the pre-decision compared to the individual condition, *t*(29) = 3.46, *p*= .002, *d*= 0.63, while the difference between the pre-decision and the final dyadic decision failed to reach significance, *t*(29) =1.79, *p*= .084 (see Table 1).

| Table 1 |  |  |
| --- | --- | --- |
| Descriptive Statistics: log *k*-value | | |
|  | *M* | *SD* |
| individual | -3.42 | 0.97 |
| pre-decision | -3.78 | 1.06 |
| dyadic | -3.87 | 1.06 |

We checked for an influence of the *order of condition* (individual first, joint first) on the log *k*-values and found an interaction effect between the *level of decision-making* and the *order of condition, F*(1.49, 41.83) = 17.85, *p*< .001, *ηp^2^*= 0.39*. We found that when conducting the individual condition first, participants in the individual condition showed higher discounting compared to both the dyadic condition, *t*(14) = 6.22, *p*< .001, *d*= 1.61, and the pre-decision, *t*(14) = 5.87, *p*< .001, *d*= 1.52. We further found lower discounting in the dyadic condition compared to the pre-decision, *t*(14) = 2.25, *p*= .041, *d*= 0.58. When conducting the joint condition first, we found no significant differences at all, all *t* < 0.28 and all *p >*.786 (see Table 2).

| Table 2 |  |  |  |  |
| --- | --- | --- | --- | --- |
| Descriptive Statistics: log *k*-value | | | |  |
|  | individual first | | joint first | |
|  | *M* | *SD* | *M* | *SD* |
| individual | -3.45 | 1.19 | -3.39 | 0.73 |
| pre-decision | -4.16 | 1.24 | -3.39 | 0.69 |
| dyadic | -4.33 | 1.18 | -3.41 | 0.69 |

In order to evaluate the statistic results, we further provided Pearson Correlations between all three levels of decision-making in terms of the percentage of SS choices and the log *k*-values (see Table 3).

| Table 3 | |  |  |  |  |  |  |  |
| --- | --- | --- | --- | --- | --- | --- | --- | --- |
| Pearson Correlations: Experiment 1, classical paradigm | | | | | |  |  |  |
|  |  |  | percentage of SS choices in % | | | log *k*-value | | |
|  |  |  | individual | pre-decision | dyadic | individual | pre-decision | dyadic |
| percentage of SS choices in % | individual | r | — |  |  |  |  |  |
|  |  | p | — |  |  |  |  |  |
|  | pre-decision | r | 0.905 | — |  |  |  |  |
|  |  | p | < .001 | — |  |  |  |  |
|  | dyadic | r | 0.899 | 0.984 | — |  |  |  |
|  |  | p | < .001 | < .001 | — |  |  |  |
| log k-value | individual | r | 0.964 | 0.843 | 0.825 | — |  |  |
|  |  | p | < .001 | < .001 | < .001 | — |  |  |
|  | pre-decision | r | 0.903 | 0.971 | 0.964 | 0.847 | — |  |
|  |  | p | < .001 | < .001 | < .001 | < .001 | — |  |
|  | dyadic | r | 0.864 | 0.951 | 0.976 | 0.798 | 0.965 | — |
|  |  | p | < .001 | < .001 | < .001 | < .001 | < .001 | — |

**Experiment 2**

We performed a repeated measures analysis of variance (ANOVA) with the factor *paradigm* (classical intertemporal choice vs. gamified intertemporal choice) and *condition* (individual condition vs. dyadic condition) on the log *k*-values, resulting in a significant main effect for paradigm, *F*(1, 29) = 95.38, *p* < .001, *ηp^2^* = 0.77, indicating that participants discounted less in the gamified version. However, the main effect for condition failed to reach significance, *F*(1, 29) = 3.93, *p* =.057, as well as the interaction between both factors, *F*(1, 29) = 0.12, *p* =.737.

Second, we performed a repeated measures ANOVA with the factors *paradigm* (classical vs. gamified) and *level of decision- making* on the difference of the log *k*-values between individual decision and pre-decision versus the difference between pre-decision and dyadic decision (see Table 4). We found no significant effect, all *F*(1, 29) < 1.02, all *p* >.322.

| Table 4 |  |  |  |  |
| --- | --- | --- | --- | --- |
| Descriptive Statistics: log *k*-value | | | | |
|  | classical | | gamified | |
|  | *M* | *SD* | *M* | *SD* |
| individual | -3.37 | 0.94 | -0.98 | 0.47 |
| pre-decision | -3.38 | 1.27 | -1.13 | 0.60 |
| dyadic | -3.50 | 1.49 | -1.17 | 0.49 |

**Exploratory analyses**

We performed a repeated a measures analyses (ANOVA) with the within-factors *paradigm* (classical, gamified) and *level of decision-making* (individual, pre-decision, dyadic decision) and the between factor *order of condition* (individual first, joint first) on the log *k*-values and found a significant interaction between all three factors, *F*(2, 56) = 3.97, *p*= .025, *ηp^2^*= 0.12. To get a deeper insight into this interaction effect, we performed post hoc comparisons for which we reduced the level of complexity stepwise, performing ANOVAs with the factors *level of decision-making* and *order of condition* similarly to Experiment 1, but separately for each paradigm, followed by *t*-tests for significant effects.

**Classical**: We found no main effect for *level of decision-making* or factor *order of condition,* all *F* < 3.19 and all *p* > .085, but a significant interaction effect between the two factors, *F*(1.75, 49.10) = 5.95, *p* = .007, *ηp^2^* = 0.17**.* To get a deeper understanding of this interaction, we conducted post hoc paired *t*-tests for each group separately, similar to Experiment 1. We found that when the joint decision-making condition was conducted first, participants performed lower discounting in the individual condition compared to the pre-decision, *t*(14) = 3.04, *p*= .009, *d*= 0.79, while all other comparisons failed to reach significance, all *t* < 1.95 and all *p* > .072. When participants performed the individual condition first, we found no effect at all, all *t* < 1.92 and all *p* > .075 (see Table 5).

**Gamified:** We found a significant main effect for level of decision-making, *F*(1.45, 40.59) = 4.92, *p* = .020, *ηp^2^* = 0.15*, and no significant effect for order of condition, *F*(1,28) = 1.59, *p* = .217, and no significant interaction, *F*(1.45, 40.59) = 0.07, *p* = .880*. Post hoc comparison revealed that the participants performed lower discounting in the dyadic condition compared to the individual condition, *t*(29) = 2.95, *p*= .006, *d* = 0.54, while all other comparison failed to reach significance, all *t <*1.90 and all *p* > .067 (see Table 5).

| Table 5 |  |  |  |  |  |  |  |  |
| --- | --- | --- | --- | --- | --- | --- | --- | --- |
| Descriptive Statistics: log *k*-value | | | |  |  |  |  |  |
|  | classical | | | | gamified | | | |
|  | individual first | | joint first | | individual first | | joint first | |
|  | *M* | *SD* | *M* | *SD* | *M* | *SD* | *M* | *SD* |
| individual | -3.51 | 0.93 | -3.23 | 0.96 | -0.86 | 0.47 | -1.11 | 0.46 |
| pre-decision | -3.87 | 1.36 | -2.89 | 0.98 | -1.02 | 0.50 | -1.23 | 0.70 |
| dyadic | -3.99 | 1.73 | -3.01 | 1.04 | -1.07 | 0.44 | -1.28 | 0.53 |

In order to evaluate the statistic results, we further provided Pearson Correlations between all three levels of decision-making in terms of the percentage of SS choices and the log *k*-value (see Table 6 for the classical and Table 7 for the gamified paradigm).

| Table 6 | |  |  |  |  |  |  |  |
| --- | --- | --- | --- | --- | --- | --- | --- | --- |
| Pearson Correlations: Experiment 2, classical paradigm | | | | | |  |  |  |
|  |  |  | percentage of SS choices in % | | | log *k*-value | | |
|  |  |  | individual | pre-decision | dyadic | individual | pre-decision | dyadic |
| percentage of SS choices in % | individual | *r* | — |  |  |  |  |  |
|  |  | *p* | — |  |  |  |  |  |
|  | pre-decision | *r* | 0.907 | — |  |  |  |  |
|  |  | *p* | < .001 | — |  |  |  |  |
|  | dyadic | *r* | 0.907 | 0.991 | — |  |  |  |
|  |  | *p* | < .001 | < .001 | — |  |  |  |
| log k-value | individual | *r* | 0.970 | 0.866 | 0.879 | — |  |  |
|  |  | *p* | < .001 | < .001 | < .001 | — |  |  |
|  | pre-decision | *r* | 0.877 | 0.985 | 0.980 | 0.839 | — |  |
|  |  | *p* | < .001 | < .001 | < .001 | < .001 | — |  |
|  | dyadic | *r* | 0.859 | 0.926 | 0.957 | 0.867 | 0.935 | — |
|  |  | *p* | < .001 | < .001 | < .001 | < .001 | < .001 | — |

| Table 7 | |  |  |  |  |  |  |  |
| --- | --- | --- | --- | --- | --- | --- | --- | --- |
| Pearson Correlations: Experiment 2, gamified paradigm | | | | | |  |  |  |
|  |  |  | percentage of SS choices in % | | | log *k*-value | | |
|  |  |  | individual | pre-decision | dyadic | individual | pre-decision | dyadic |
| percentage of SS choices in % | individual | *r* | — |  |  |  |  |  |
|  |  | *p* | — |  |  |  |  |  |
|  | pre-decision | *r* | 0.814 | — |  |  |  |  |
|  |  | *p* | < .001 | — |  |  |  |  |
|  | dyadic | *r* | 0.832 | 0.974 | — |  |  |  |
|  |  | *p* | < .001 | < .001 | — |  |  |  |
| log k-value | individual | *r* | 0.840 | 0.740 | 0.742 | — |  |  |
|  |  | *p* | < .001 | < .001 | < .001 | — |  |  |
|  | pre-decision | *r* | 0.806 | 0.937 | 0.901 | 0.728 | — |  |
|  |  | *p* | < .001 | < .001 | < .001 | < .001 | — |  |
|  | dyadic | *r* | 0.820 | 0.943 | 0.931 | 0.732 | 0.938 | — |
|  |  | *p* | < .001 | < .001 | < .001 | < .001 | < .001 | — |

1. Kirby, K. N., Petry, N. M., & Bickel, W. K. (1999). Heroin addicts have higher discount rates for delayed rewards than non-drug-using controls. *Journal of Experimental Psychology. General*, *128*(1), 78–87. [↑](#footnote-ref-1)
