# Supplementary material for: Effects of individual and dyadic decision-making and normative reference on delay discounting decisions
Source: Cogn Res Princ Implic. 2022 Jul 28;7:71. doi: 10.1186/s41235-022-00422-5 (PMC9334506; doi:10.1186/s41235-022-00422-5)
Supplement: Supplementary file 3 — Additional file 3 Results of the main analyses with gradual inclusion of participants previously excluded due to very strong or weak discounting. [file 41235_2022_422_MOESM3_ESM.docx]

**Effects of individual and dyadic decision making and normative reference on delay discounting decisions**

Supplement Materials S3

# Diana Schwenke, Peggy Wehner, Stefan Scherbaum

# Department of Psychology, Technische Universität Dresden, Dresden, Germany

In our study, participants with a relative frequency of SS choices of more than 80% or less than 20% in the individual condition in any of the two paradigms were completely excluded from the analysis. If at least one of the two participants within a pair met the exclusion condition, the entire pair was excluded. We used this approach to avoid any ceiling or floor effects regarding the individual condition but also to ensure that we did not produce any artificial effect due to regression the mean.

In this section of the supplementary material we analyzed how the results of the main analyzes of interest change when the excluded pairs of participants are gradually added to the analyzes.

To gradually add the excluded pairs of participants to the analyzes, we have changed the cutoff points of the exclusion step by step. The starting point of all analyzes was the sample of pairs of participants from the analyzes of the result section of the main manuscript with the cutoff points of the relative frequency of choosing the SS option of at least 20% and at most 80% in the individual condition. These cutoff points were gradually increased or decreased by 5% so that in the next step both participants in a pair had a relative frequency of SS choice between 15% and 85%, in the next step between 10% and 90%, then between 5% and 95% and in the last step, all recorded pairs of participants were included in the analyzes. The main analyzes of interest were then carried out for all 5 samples.

**Experiment 1**

In Experiment 1, the analyzes dealt with the questions whether the choices in the levels of decision (individual decision, pre-decision, dyadic decision) differ from one another and whether there are differences depending on the order of condition (individual first, joint first).

In order to check whether the levels of decision differed, we performed paired *t*-tests on relative frequency of SS choices between all levels of decision. We carried out these analyzes both for all pairs of participants, regardless of the order of conditions, and separately according to the order of conditions. To investigate an interaction effect between the order of conditions and the levels of decision, we performed independent *t*-tests in which we compared the differences of two levels of decision between the conditions *individual first* and *joint first*.

The resulting *p*-values of all main analyzes of interest are summarized for all 5 samples in Figure 1.


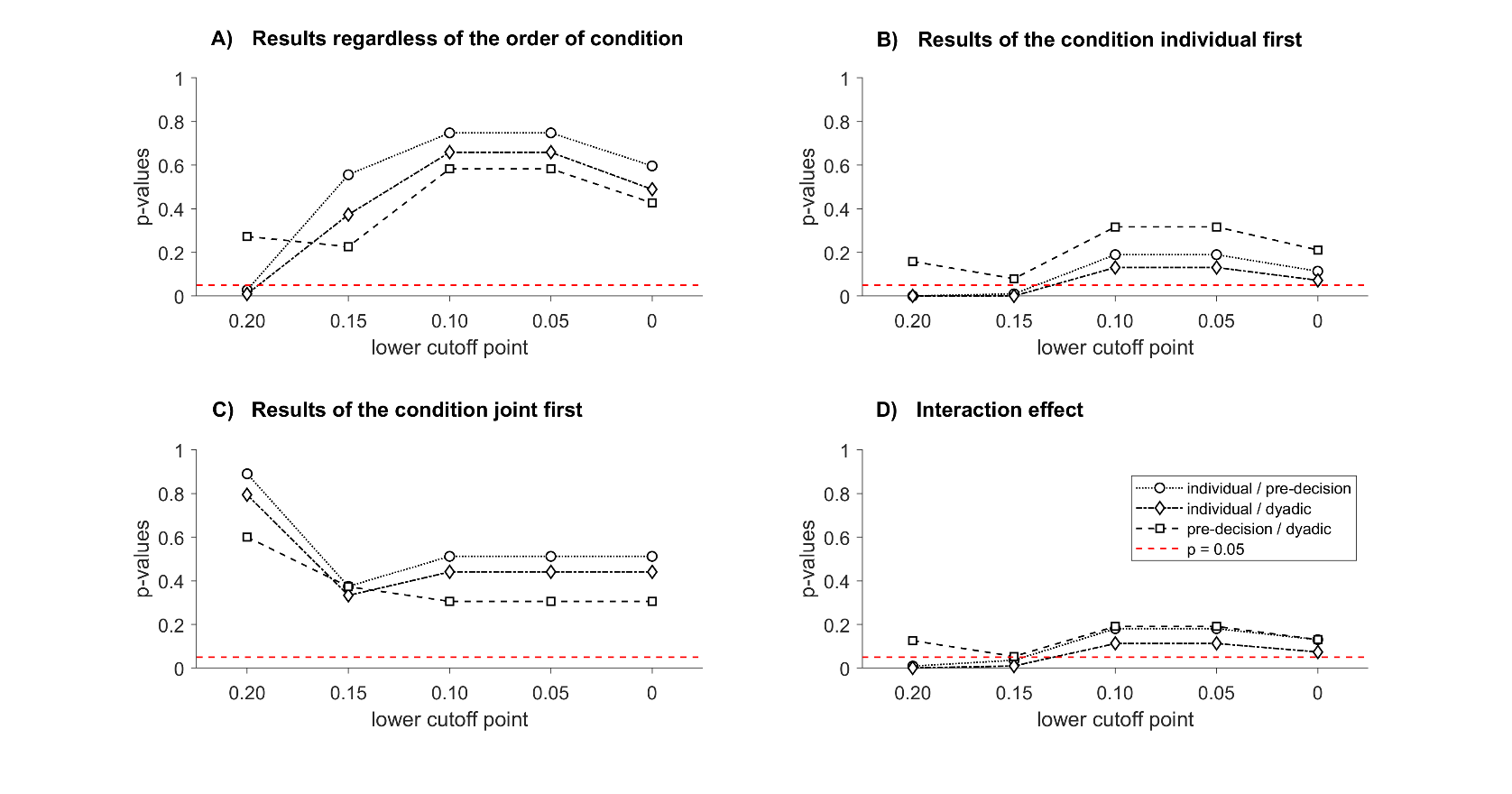


***Fig. 1 Changes in p-values and of the main analyzes of interest of Experiment 1 depending on the inclusion of outliers.*** *The p-values of the main analyzes change due to the inclusion of pairs of participants who were excluded from the original analyzes. The abscissa axis indicates the minimum relative frequency with which the SS option had to be chosen so that the participants were included in the analyzes. It thus represents the lower cutoff point. The upper cutoff point of the inclusion is the difference between 100 and the lower cutoff point. (A) The analyzes of all pairs of participants, regardless of the order of conditions, and (B) the analyzes of pairs of participants who conducted the individual condition first showed that there was a significant difference between the individual condition and the pre-decision before the inclusion of former excluded dyads. These differences are no longer significant after the inclusion of former excluded pairs of participants. (C) In the analyzes of the pairs of participants who conducted the dyadic condition first, we did not find any significant results regardless of the inclusion of the former excluded dyads. (D) The significance of the interaction effect between order of condition and level of decision thus also changes due to the inclusion of former excluded dyads.*  **Experiment 2**

In Experiment 2, the analyzes also dealt with the questions whether the choices in the levels of decision (individual decision, pre-decision, dyadic decision) differ from one another and whether there are differences depending on the order of condition (individual first, joint first). We also analyzed the results separately for the classical paradigm and the gamified paradigm.

To check whether the levels of decision differed in the classical paradigm, we performed paired *t*-test on relative frequency of SS choices between all levels of decision. We carried out these analyzes separately for the pairs of participants who conducted the individual condition first and for the pairs of participants who conducted the joint condition first. In order to investigate an interaction effect between the order of conditions and the levels of decision in the classical paradigm, we performed independent *t*-tests in which we compared the differences of two levels of decision between the conditions *individual first* and *joint first*.

Since performing an ANOVA with the factors *level of decision* and *order of condition* in the gamified paradigm, both before the inclusion of excluded dyads, *F*(1.23, 34.46) = 0.16, *p* = .745 (Greenhouse-Geisser corrected), and after the inclusion of these dyads, *F*(1.27, 47.14) = 0.56, *p* = .498 (Greenhouse-Geisser corrected), did not reveal a significant interaction effect between the two factors, we analyzed all pairs of participants together in this paradigm regardless of the *order of condition*. In order to check whether the levels of decision differed, we performed paired *t*-tests on relative frequency of SS choices between all levels of decision.

The resulting *p*-values of these main analyzes of interest are summarized for all 5 samples in Figure 2.
**
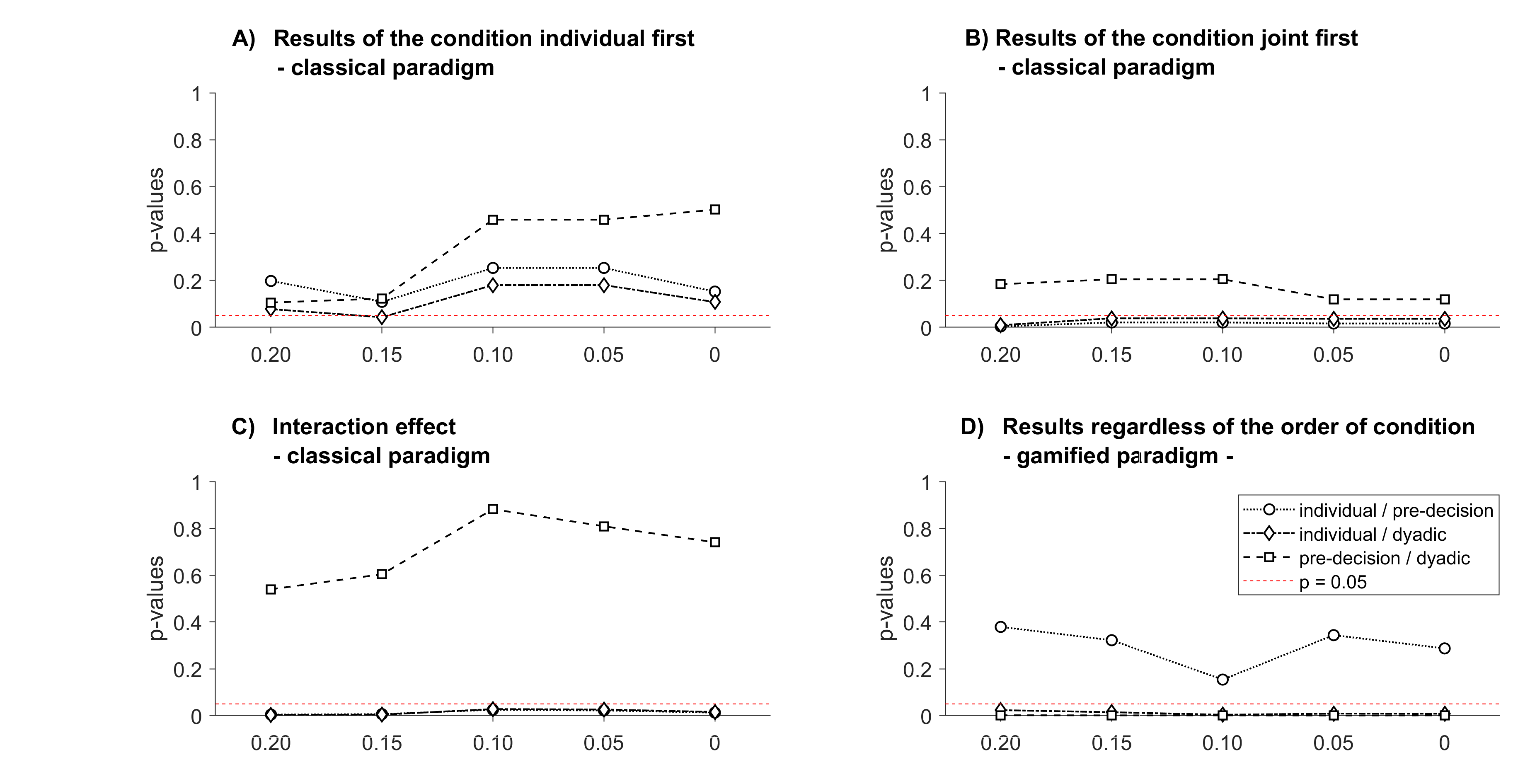
**

***Fig. 2 Changes in p-values of the main analyzes of interest of Experiment 2 depending on the inclusion of outliers.*** *The p-values of the main analyzes change due to the inclusion of pairs of participants who were excluded from the original analyzes. The abscissa axis indicates the minimum relative frequency with which the SS option had to be chosen so that the participants were included in the analyzes. It thus represents the lower cutoff point. The upper cutoff point of the inclusion is the difference between 100 and the lower cutoff point. In the main analyzes of Experiment 2, the inclusion of former excluded dyads did not change the significance of the results. (A) In the classical paradigm, we did not find any significant results in the analyzes of the pairs of participants who conducted the dyadic condition first. (B) The analyzes of pairs of participants who conducted the dyadic condition first showed that there were significant differences between the individual condition and the pre-decision and between the individual condition and the dyadic condition. (C) The interaction effect between order of condition and level of decision was thus significant for the differences of the individual condition and the pre-decision and the differences of the individual condition and the dyadic condition. (D) In the gamified paradigm, we found significant differences between the pre-decision and the dyadic condition and between the individual condition and the dyadic condition.*

**Conclusion**

In our study, participants with a relative frequency of SS choices of more than 80% or less than 20% in the individual condition were excluded from the analysis. We used this approach to avoid any ceiling or floor effects but also to ensure that we did not produce any artificial effect due to regression to the mean. In this section of the supplementary material we presented how the results of the main analyzes of interest change when the excluded dyads are gradually added to the analyzes.

In Experiment 1, we found no more significant differences in various analyzes in which we found significant results prior to the inclusion of the originally excluded dyads. Our results in Experiment 1 were very susceptible to interference, even without the inclusion of the excluded dyads, so that this result is hardly surprising. We investigated these uncertainties in more detail by carrying out the preregistered Experiment 2. In the main analyzes of interest of Experiment 2, the inclusion of excluded dyads did not change the significance of the results. Therefore, the results of this experiment are robust against different inclusion and exclusion criteria of participants.
