# Supplementary material for: Effects of individual and dyadic decision-making and normative reference on delay discounting decisions
Source: Cogn Res Princ Implic. 2022 Jul 28;7:71. doi: 10.1186/s41235-022-00422-5 (PMC9334506; doi:10.1186/s41235-022-00422-5)
Supplement: Supplementary file 4 — Additional file 4: Analysis of the factors influencing the participants' behavior in the pre-decision. [file 41235_2022_422_MOESM4_ESM.docx]

**Effects of individual and dyadic decision making and normative reference on delay discounting decisions**

Supplement Materials S4

# Diana Schwenke, Peggy Wehner, Stefan Scherbaum

# Department of Psychology, Technische Universität Dresden, Dresden, Germany

In our experiments, the participants executed a series of choices between two delayed options in an individual (*individual condition*) and in a joint decision making condition (*dyadic decision*) using a non-verbal choice selection procedure. In the individual condition, each participant moved a curser into an option associated response box via joystick movement. In the dyadic decision, both joystick movements were added, so that both participants controlled the cursor together and had to coordinate in order to reach a mutual decision. Importantly, the cursor only began to move after both participants indicated their first initial choice preference. With the help of this procedure, we separated the final dyadic choice from each participant’s initial individual preference. This individual decision was made before the partner’s preference in the current trial was known. We call this initial preference of each participant the *pre-decision*.

However, it is an open question which factors influence the behaviour of the participants in the pre-decision. Our assumption is that during the pre-decision, the presence of a partner creates a social context which, for example, could lead to a reduced discounting behaviour of the participants. Since our experiments consist of series with a large number of trials, it would also be possible that what a participant learns about the partner’s preference in the first trials influences his behaviour in the following trials. If of two participants in a dyad, one participant has a consistent preference for one of the two options, it is particularly easy for the other participant to quickly identify the partner’s preference and to adapt his behaviour accordingly. If a participant does not have a consistent preference, then this is much more difficult.

In order to get a sense for how often the participants had a consistent preference for an option, we took a closer look at the relative frequencies of the SS choices in the pre-decision over the course of the experiments or paradigms.

To examine in more detail the possibility that the participants adapt to the partner’s preference over the course of the experiments already in the pre-decision, we also analyzed whether the difference in the relative frequency of SS choices between the participants of a dyad changes over time. For this purpose, we have divided our series of trials separately for each experiment and paradigm into groups of 10 trials each. If the number of trials differed between the dyads, we used the minimum number of trials within an experiment or paradigm. For each group of trials, we determined the difference in the relative frequency of SS choices between the participants of a dyad.

For each experiment or paradigm, we first performed a repeated measures analysis of variance on the determined differences with the repeated measures factor *time course* (the number of levels corresponds to the number of determined trial groups). In addition, we determined a linear, quadratic and cubic contrast for each experiment or paradigm to identify any form of trend in the data. We also calculated the repeated contrasts to analyze how the differences in the relative frequency of SS choices differ in two consecutive groups of trials.

**Consistent preferences**

Figure 1 shows the relative frequency of SS choices within the first 5, 15, 25, 35 and 45 trials in box plots separately for Experiment 1, the classical paradigm of Experiment 2 and the gamified paradigm of Experiment 2.
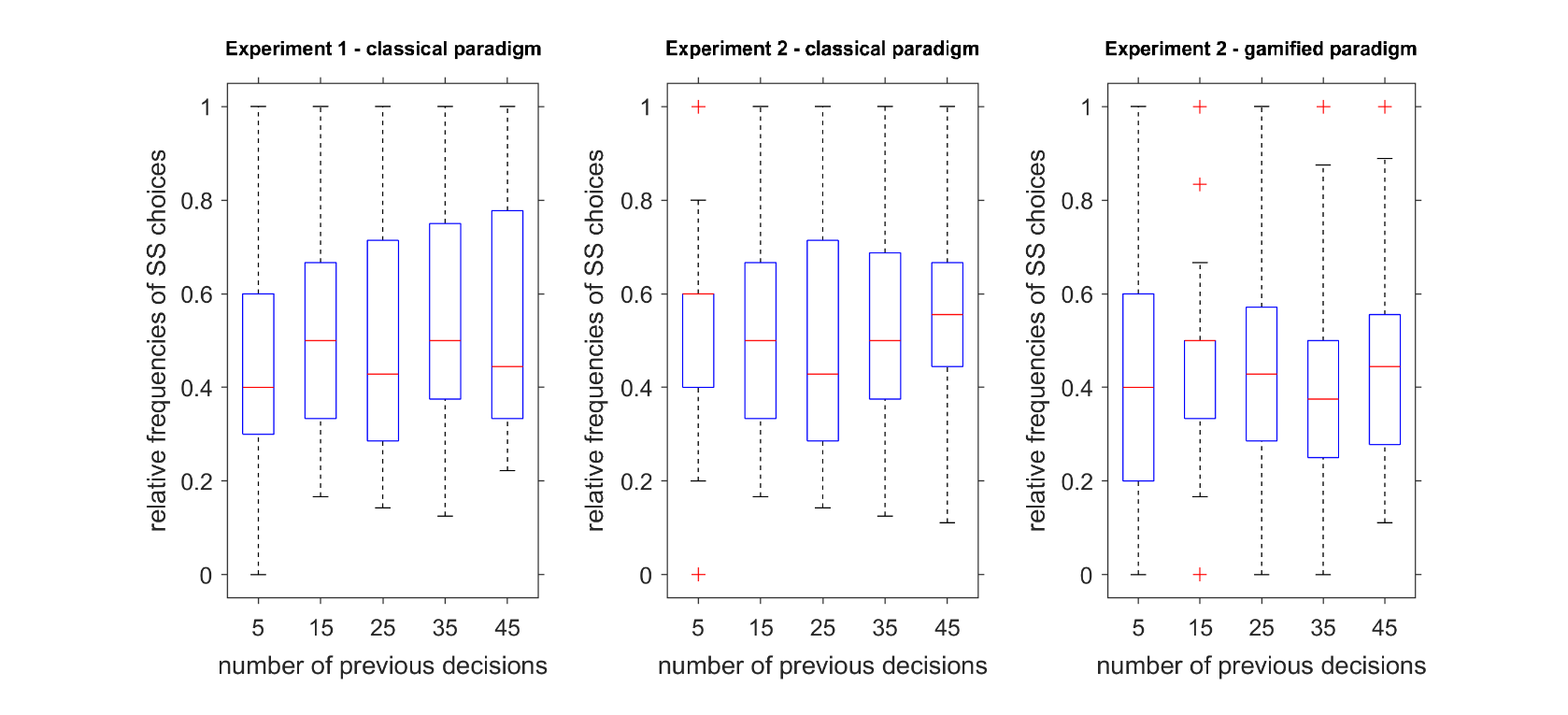


***Fig. 1 Box plots of the relative frequencies of SS choices in the first trials of an experiment or paradigm.*** *The illustration of the relative frequencies of SS choices in the pre-decision condition in the first 5, 15, 25, 35 and 45 trials of an experiment or paradigm shows that the majority of the participants did not have a consistent preference for one of the two options within the first trials. The majority of the participants chose both options with similar frequency.*

**Adaptation of behavior to the partner’s preference over the course of an experiment**

In Experiment 1, the analysis of the differences in the relative frequency of SS choices between the participants of a dyad in the examined trial groups did not reveal a significant main effect of the factor *time course*, *F*(21, 609) = 1.20, *p* = .248. The determination of a linear, *F*(1, 29) = 2.41, *p* = .131, quadratic, *F*(1, 29) = 0.24, *p* = .626, and cubic contrast, *F*(1, 29) = 3.41, *p* = .075, also showed no significant result. The results of the paired *t*-tests between two trial groups that followed one another over time were also not significant, all *t* < 2.01 and all *p* > .054.

In the classical paradigm of Experiment 2, the repeated measures ANOVA also showed no significant main effect of the factor *time course*, *F*(21, 609) = 1.52, *p* = .064. The determination of linear, *F*(1, 29) = 0.10, *p* = .754, quadratic, *F*(1, 29) = 4.14, *p* = .051, and cubic contrasts, *F*(1, 29) = 1.11, *p* = .302, also did not lead to significant results. The results of the paired *t*-tests between two trial groups that followed one another over time were also not significant, all *t* < 1.94 and all *p* > .062.

In the gamified paradigm of Experiment 2, the repeated measures ANOVA also showed no significant main effect of the factor *time course*, *F*(11, 319) = 0.73, *p* = .705. The determination of linear, *F*(1, 29) = 4.09, *p* = .052, quadratic, *F*(1, 29) = 0.48, *p* = .493, and cubic contrasts, *F*(1, 29) = 0.164, *p* = .688, also did not lead to significant results. The results of the paired *t*-tests between two trial groups that followed one another over time were also not significant, all *t* < 1.33 and all *p* > .194.

**Conclusion**

It cannot be seen from the data that the majority of the participants already had a consistent preference for the SS or LL option at the beginning of an experiment or paradigm. In our opinion, it is therefore unlikely that in the majority of dyads it was relatively easy for a participant to identify the partner’s preference within a few trials and to adapt to this behavior.

In addition, since the analyzes of the differences in the relative frequency of SS choices between the participants of a dyad in the examined trial groups do not show a significant main effect of the factor *time course* in any experiment or paradigm, we do not assume that the pre-decisions of the participants in a dyad over the trial series will adapt to one other. This assumption is also supported by the analyzes of the contrasts. We therefore believe that we can capture the individual preference of a participant in a social context through the pre-decision of our design.

Obviously, despite these results, we cannot assume that the reduction in discounting in the pre-decision compared to the individual condition can only be attributed to the existence of a social context. The choices in the pre-decision can of course also be influenced by other factors. However, we cannot confirm a systematic effect of the adaptation to the partner over time.
